# [Retracted] Radioprotective effects of pyrroloquinoline quinone on parotid glands in C57BL/6J mice

**DOI:** 10.3892/etm.2024.12644

**Published:** 2024-07-08

**Authors:** Yuanqing Huang, Ning Chen, Dengshun Miao

Exp Ther Med 12:3685–3693, 2016; DOI: 10.3892/etm.2016.3843

Following the publication of this paper, a concerned reader drew to the Editor’s attention that the mice featured in Fig. 1A on p. 3687 were strikingly similar to mice that had already been published in Fig. 1A of a different paper (doi: 10.3892/etm.2015.2532) written by the same research group in the same journal, although the experiments were described differently. In spite of the similarity in the appearance of the mice, however, the images in the above paper appeared to have undergone some editing, as the animals were shown of different relative sizes, in different relative positions, and possible splice marks in the background were also visible.

In view of the apparent anomalies that have been identified, the Editor of *Experimental and Therapeutic Medicine* has decided that the above paper should be retracted from the Journal. After having been in contact with the authors, they accepted the decision to retract the paper. The Editor apologizes to the readership for any inconvenience caused.

